# Letters on Yellow Fever at Memphis, Tenn., in 1853

**Published:** 1854-04

**Authors:** Geo. A. Smith, W. J. Tuck

**Affiliations:** Physician to the Memphis Hospital; Memphis, Tenn.; Memphis; Memphis, Tenn.


From the New Orleans Med. and Surg. Journal.

*Letters on Yellow Fever at Memphis, Tenn., in* 1853. By Geo. A. Smith, M. D., Physician to the Memphis Hospital, and W. J. Tuck, M. D., of Memphis.

(We commend the following letters to the special attention of our readers. *Ed.*)

Memphis, Tenn., Dec., 1853.

*Editor N. O. Med. and Surgical Journal.*

Dear Sir—With the belief that it may be a matter of some interest to trace the phenomena of Yellow Fever when carried away from the head quarters of its epidemic fury, the following remarks have been written upon some cases which were brought for treatment to the Memphis Hospital.

The city of Memphis is well known to be by river about 800 miles above New Orleans, and is the lowest point of any considerable population which escaped the fearful epidemic of 1853, having been not only free from Yellow fever, but favored with a much healthier summer and autumn than has fallen to its lot for several years. The mortality from May to August, inclusive, (generally the unhealthy season here) was in 1853 considerably less than half what it had been two preceding years.

The nearest point to Memphis at which Yellow Fever prevailed as an epidemic was Napoleon, Arkansas, about 200 miles below; and it ascended to no point above the city; so that Memphis was the lowest point on the Mississippi river, of any considerable population, which enjoyed this happy immunity. On the other hand, it prevailed with virulence at Natchez, Vicksburg, and several of the smaller towns intermediate.

All the cases which were brought to the Memphis Hospital, in number 62, were landed from the river, and contracted the disease in New Orleans and its immediate vicinity. The mortality, as might be expected was very great, from the fact that they were not landed till the disease had taken full possossion of the system, and then carried the distance of a mile from the wharf-boat to the Hospital, on an open dray. Of the 36 who died, 17 lived less than twelve hours after their arrival.

Three methods of treatment were adopted, of which the most successful, in fact the only successful one, was the following: On their arrival, after a hot mustard foot-bath and being warmly covered in bed, a laxative, if necessary, was administered, of an infusion of renna, rhubarb and manna; cups and mustard to epigastrium, with ice given solid and iced mucilage; opiates, particularly the Black Drop, and milk punch ad libitum. Several cases were cured under this treatment, after the black vomit had been well established. The other methods tried were that of calomel and opium in broken doses, with twenty drops of laudanum—both entirely without success.

The question of the contagiousness of Yellow Fever was fully tested, and the result confirms the impression, that without the pre-requisite conditions of atmospheric and climatic derangement, Yellow Fever is no more liable to be communicated from one subject to another than gout or rhematism. At any rate the 62 patients who were treated in the Memphis Hospital were all of them distributed promiscuously through the wards of the institution; treated there throughout their progress to recovery or death, and no single instance occurred of the disease upon them.

The above brief statement is respectfully submitted to you. In relation to so terrible a scourge as the epidemic of 1853, it is presumed that as large an accumulation of facts as possible is most desirable—facts derived from observation made under all possible circumstances; and provided they are plainly and truthfully stated, ought to be acceptable, whatever the sources from which they are derived. The circumstances under which my observations were made were in this respect peculiar, indeed almost unique, as Memphis was probably the only place where a considerable number of *imported cases* could have been treated, in a region itself exempt from epidemic influences.

Respectfully, GEO. A. SMITH.

REMARK.—We are at a loss to conceive how any one could expect benefit from doses of 20 grs. quinine and 20 drops laudanum in the advanced stages of Yellow Fever—say the third or fourth day. We have seen no such treatment recommended by any advocate of quinine, but on the contrary, always forbidden. Calomel and opium in this stage is not much better. F., *Ed.*

---

*Editor N. O. Med. and Surgical Journal.*

MEMPHIS, Tenn., March 19, 1853.

MY DEAR SIR—I applied a few days since to Dr. Smith, Physician of the Hospital here, in relation to the subject of your enquiry, and he informed me that he had recently transmitted all the statistical information connected with the subject to Dr. Hester, and which probably reached its destination about the time of his death. The Doctor wished me to refer you that letter. He informed me at the same time that there were 62 cases of Yellow Fever admitted into the Hospital, which were taken from the boats landing at this place and 36 deaths, and that in no instance was the disease communicated to others. As well as my memory serves me, we had only three cases of Yellow Fever in this city; two of these had been exposed to the epidemic in New Orleans, and probably contracted the seeds of the disease there; the third was a citizen of our place and had not been absent, and it was regarded at the time as a sporadic case, such as occasionally occurs in this city.

You request me to give my view in regard to the contagiousness of Yellow Fever. So far as I have been competent to investigate this subject, and so far as my own observation extends, I am a decided noncontagionist. During my residence in New Orleans, in the summer of 1841, and while one of the attending Physicians of the Charity Hospital during the epidemic of that summer, I think it was the unanimous sentiment of all the prominent physicians of the city that the disease was not contagious, nor do I think any one pretended at that time that the epidemic of that season was imported from abroad. Such, I believe, have been your own statements, as evinced in your writings on this subject. Whether the opinions of the physicians of your city have undergone any change in relation to this matter since the breaking out of the late epidemic, I have not been able to gather any accurate information.

Still the intimation has been thrown out, that circumstances have been developed in connexion with the recent epidemic, which have led some to alter their opinions and to favor their doctrine of contagion. However that may be, we are all perfectly well satisfied *here* that the disease could not be communicated by contagion in the atmosphere of our city; and it seems to me, if I understand the meaning of the word contagion, in its proper acceptation, that contagious diseases may be communicated and reproduced from one person to another, without any regard to the temperature or latitude; as for instance, small-pox, scarlet fever, measles, etc.

Now, let us look for a moment to the facts of the case in our city. We adopted no quarantine regulations; we had instituted no especial sanitary regulations with the view of preventing an incursion of the epidemic. Boats freighted with merchandize, saturated with the atmosphere of an infected city, landed at our wharf almost every day; a number of persons affected with the disease were carried through our streets to the Hospital, and some of them dying in private families, and not a case was communicated to any of the nurses, friends or physicians.

It certainly must be a very singular and unprecedented sort of contagious disease which could not be communicated in a single instance, under such favorable circumstances! when the streets of our city were as filthy as usual; when the weather was very warm; when there was no avoidance of exposure, and when every condition existed to promote contagiousness! Does any person suppose for a moment, that there could be brought into our city such a number of cases of any of those diseases ordinarily termed contagious, without the occurrence of a single instance of communication to the unprotected and exposed? During the month of September it fell to my lot to be called upon professionally to attend some cases of Yellow Fever occurring on the steamer H. D. Bacon, from New Orleans. When the boat arrived here some ten cases were taken off and sent to our Hospital; I was retained professionally to attend some of the officers to their point of destination, St. Louis. I must confess that I had some apprehension of danger when I first went on board of the boat; not from fear of contagion, but from the infected atmosphere, which I supposed might still have remained to some extent. The boat was a large one, heavily freighted with merchandize, and a large number of the crew and passengers were sick, some of whom died before reaching here. Here, certainly, was as fine and fair an opportunity for an unacclimated and unprotected person to contract a contagious disease as we can well suppose to exist; and yet what are the facts of the case? Not a passenger, out of some dozen or more, who took passage on the boat, above the infected region, suffered from any symptom of the disease. And yet the second clerk, and the captain, who was on duty until he reached St. Louis, had contracted the seeds of the disease in New Orleans, and after I returned home to Memphis I learned that both had died of Yellow Fever several days after their arrival at St. Louis. I am happy to state that the several cases which were under my care were convalescent when I reached St. Louis, and I returned immediately home; and although exposed constantly day and night to whatever source of contagion there might have been, yet my health remained not only unaffected, but was better than usual.

It is said that the epidemic which has swept with so much fatality over the fairest regions of our southern country, differs in some respects from the preceding epidemics. We are disposed to believe the difference exists rather in degree than in kind. The cases we have seen here, imported from your city, have been just the same in symptoms as those we witnessed in New Orleans in 1841. It is true, the recent epidemic has been much more malignant and fatal than any of the previous ones; and it is stated that many more of those supposed to be acclimated were affected with the disease than at any previous period; but the main peculiarliy that has been insisted upon is, that the epidemic extended over a much larger scope of country than it has been previously known to do, and occurred in many towns and villages where it was never known before to exist; thus appearing to favor the opinion entertained by some, that the disease was transmitted to those localities by contagious influence, and not originating from local causes.

Now it is a very important question to determine, whether, in those cases, the disease did *originate* in certain localities, or was transmitted by persons affected with the disease, steamboats, etc.

We have long since learned enough of luman nature to know that the citizens of any particular town or locality are very slow to acknowledge their location a sickly or unhealthy one; and this is very natural; and no doubt many of the citizens of various villages in Louisiana and Mssissippi are disposed to attribute the visitation of the epidemic among them to importation from New-Orleans. Natchez has been in the habit of doing this for the last thirty years; and yet with all her quarantine regulations, as likewise those of Vicksburg, the disease has prevailed in those places with as much or more fatality than in New Orleans. It is true the quarantine regulations were not as rigid as they might have been; yet if we are correctly informed no steamboats from New Orleans were permitted to land, nor were any of the sick on those boats taken care of until the epidemic made its appearance in those towns. Now compare this condition of, things with that which existed in Memphis, not much more than a day's travel on a fast boat. Here we imposed no restrictions—had no quarantine. As far mentioned, large quantities of goods, which had been remaining for weeks in New Orleans, were daily put off at our wharf and distributed through the city; many of these goods of a molient character, well calculated to act as fomites to convey contagious effluvia; and moreover, a number of persons affected with Yellow Fever, daily taken from the boats and dying within the precincts of the city, and yet not a single instance of its communication occurring.

But the enquiry is raised, if the disease was not communicated or transmitted from one point to another, in the region South of us, why did it occur and how did it originate in some of those localities hitherto regarded as healthy and where the epidemic was never before known to exist? We reply to this by asking, how did it originate in New Orleans, Mobile and other places, at the time preceding which the epidemic was never known to occur in those latitudes? The fact is, a combination of *local causes*, we believe, sprang up at that time; which combination had never existed previously.

We are very much inclined to the opinion, that there existed in the whole region of country embraced within a certain line of latitude South of us, a *certain predisposing epidemic constitution of atmosphere*, which aided in giving rise to so extensive an epidemic. We believe that in the same way there is a predisposing and peculiar constitution of atmosphere prevailing when cholera appears in our country, only the letter is more extensive, and is but little influenced by climate, whereas the former is confined almost exclusively to hot latitudes. The probability is that the increased fatality and malignity of the epidemic this year was the result of a greater degree of this predisposing influence in the atmosphere constitution. This being the case, and other causes, such as animal or vegetable miasm, being the same as in previous epidemics, we may readily explain why the recent one should have been more fatal and malignant as well as extensive in its scope. Now supposing this predisposing atmospheric constitution to exist throughout the whole of a certain region of a warm climate, may we not thus account for the development of the disease in certain localities where it has never occurred before, without resorting to the doctrine of communication or contagion?

This opinion would seem to be corroborated by the fact, that in some of these localities it has been found impossible, from the best information we can obtain, to trace out the least communication of any kind with those towns or places where the epidemic was prevailing. And may we not in this way account for the exemption of our city and that of all other towns on the river north of a certain latitude, say that of Napoleon or Lake Providence? In this city surely we had enough of animal and vegetable filth to produce disease, as well as every circumstance to favor contagion, but we did not have the predisposing constitution of atmosphore. We are therefore disposed to believe that the disease is of local origin and not contagious; and we offer the above theory as a sufficient one, we believe, to account for the extension and prevalence of the disease in Southern localities not hitherto subject to it, without the necessity of resorting to the doctrine of contagion, importation or transmission.

Yours, respectfully,

W. J. TUCK.

